# The impact of traumatic childhood events on functioning in patients with schizophrenia

**DOI:** 10.1192/j.eurpsy.2022.1986

**Published:** 2022-09-01

**Authors:** C. Neily, N. Charfi, G. Smaoui, R. Feki, S. Omri, L. Zouari, J. Ben Thabet, M. Maâlejboauli, M. Maalej

**Affiliations:** Hedi Chaker hospital, Psychiatry Department, SFAX, Tunisia

**Keywords:** adverse childhood experiences, schizophrénia

## Abstract

**Introduction:**

A history of adverse childhood experiences (ACEs) can increase the risk of schizophrenia spectrum disorders and might be related to unfavorable clinical and functional outcomes of psychosis

**Objectives:**

To assess the relationship between the history of ACEs and functioning in stabilized patients with schizophrenia or schizoaffective disorder.

**Methods:**

We conducted a cross-sectional, descriptive and analytical study. It was carried out on out patients with stabilized schizophrenia or schizoaffective disorder. The diagnosis of schizophrenia and schizoaffective disorder was established based on DSM-5 criteria. We used the ACEs scale to screen for traumatic events that occurred in the childhood and we used the Functional Assessment Staging Scale (FAST) to assess the patients’ ability to function and perform tasks of daily living

**Results:**

Seventy five patients were included.The mean age was 39.81 ±9.96 years.The sex ratio was 4 .34. The mean score of ACE was 3.55 ± 2.41 and 88% of patients had experienced at least one traumatic event.The mean sore of the FAST scale was 33 ± 14.95.The total score of FAST was significantly higher in case of physical negligence in childhood (p=0.018). No correlation was found with the others ACEs.The FAST sub score of cognitive functioning correlated with the history of parents separation (p= 0.47) and physical negligence (p= 0.03). we also found that The FAST sub score of interpersonal relationships correlated with the history of emotional abuse (p=0.021)

**Conclusions:**

Our data has shown that ACEs contribute to functioning impairment in schizophrenia and schizoaffective disorder.This impairment affects mainly the cognitive functioning and the interpersonal relationships

**Disclosure:**

No significant relationships.

